# The impact of comorbidities on the efficacy of IL-6 inhibitor olokizumab compared to adalimumab in a randomized controlled trial

**DOI:** 10.1186/s13075-025-03682-w

**Published:** 2025-11-14

**Authors:** Eugen Feist, Michael E. Luggen, Evgeny L. Nasonov, Sergey S. Yakushin, Daria V. Bukhanova, Alina N. Egorova, Sergey A. Grishin, Mikhail Y. Samsonov, Josef S. Smolen

**Affiliations:** 1 Experimental Rheumatology, Otto-von-Guericke University, Magdeburg, Germany; 2 University of Cincinnati College of Medicine, Cincinnati, OH USA; 3 V.A. Nasonova Research Institute of Rheumatology, Moscow, Russia; 4 Ryazan State Medical University named after academician I P Pavlov, Ryazan, Russia; 5 R-Pharm JSC, Moscow, Russia; 6 Medical University of Vienna, Vienna, Austria

**Keywords:** Rheumatoid arthritis, Biological treatments, BDMARD, Adalimumab, Olokizumab, Comorbidity

## Abstract

**Background:**

Patients with rheumatoid arthritis (RA) have an increased prevalence of comorbidities, which is associated with higher RA disease activity and worse disease outcomes. The aim of this analysis was to evaluate the impact of the comorbidity burden on the efficacy of the IL-6 inhibitor olokizumab (OKZ) and the tumour necrosis factor (TNF) inhibitor adalimumab (ADA) in the CREDO-2 randomized controlled clinical trial cohort of patients with active RA.

**Methods:**

A total of 1402 patients with RA were included in the analysis and divided into two groups on the basis of the modified Charlson Comorbidity Index (mCCI) at baseline: those having no comorbid conditions, NCC (mCCI = 1; RA only) vs. those having comorbid conditions, CC (mCCI ≥ 2; RA and ≥ 1 comorbidity). The key outcomes at Week (W) 24 were the proportions of patients with CDAI ≤ 10 and CDAI ≤ 2.8, other outcomes were ACR50 (W12, W24), proportions of patients with SDAI ≤ 3.3 (W24).

**Results:**

All groups had similar proportions of approximately 25% of patients with mCCI ≥ 2. There was no significant difference in efficacy between the OKZ q4w or q2w-treated NCC and CC groups at 3 and 6 months of treatment. The same was observed for the placebo group. In contrast, comorbidities reduced CDAI ≤ 10 and ACR50 outcomes upon ADA treatment at 6 months.

**Conclusion:**

This post hoc analysis of the phase III CREDO-2 study suggests that the presence of at least one CCI comorbidity, including common disorders such as chronic pulmonary diseases and cardiovascular diseases, does not affect the OKZ treatment results in RA patients. In contrast, comorbidities reduce several efficacy outcomes upon ADA treatment at 6 months. The CCI was not associated with placebo group results and had no influence on safety outcomes.

**Trial registration:**

NCT02760407 submitted 2016-05-02.

**Supplementary Information:**

The online version contains supplementary material available at 10.1186/s13075-025-03682-w.

## Background

Rheumatoid arthritis (RA) is associated with increased rates of comorbid conditions, such as cardiovascular and pulmonary diseases, malignancies and infections [1]. The presence of comorbidities is associated with increased RA activity, greater functional impairment, reduced quality of life, and increased mortality [2–6]. The main goal of RA treatment is to achieve remission (REM) or low disease activity (LDA) [7]. Currently, more than ten biologic (b) disease-modifying antirheumatic drugs (DMARDs) and targeted synthetic (ts) DMARDs of different classes and mechanisms of action are used for RA treatment, which raises the important issue of selecting the appropriate therapy for each patient with active RA when conventional synthetic (cs) DMARDs are not sufficiently effective. Analysis of the COMORA registry [8] revealed that disease activity, as per the Disease Activity Score using 28 joint counts (DAS28), was significantly higher in patients with RA and concomitant comorbidities, such as diabetes mellitus, coronary heart disease, and obesity. Additionally, in an analysis of the CORRONA registry conducted in 2013 [9], compared with the absence of comorbidities, comorbidities were associated with a lower likelihood of achieving RA remission according to the stringent criteria of the clinical disease activity index (CDAI) (*p* < 0.001). Although the impact of comorbidities on the effectiveness of RA therapy is an important issue and is being studied, there are still insufficient data on the influence of comorbidities on the efficacy and safety of separate bDMARDs classes.

For a personalized approach to RA treatment, it is important to evaluate potential predictors of response to a specific bDMARD. This particularly pertains to patients with comorbidities for whom finding an optimal strategy may be especially challenging.

The post hoc analysis of the CREDO-2 study presented here aimed at precisely investigating this aspect, namely, the impact of comorbidities on the efficacy and safety of olokizumab (OKZ) [10–13], a humanized monoclonal antibody (mAb) developed as an inhibitor of interleukin-6 (IL-6), and adalimumab (ADA), an anti-TNF agent, in patients with RA and an inadequate response to csDMARDs who were included in this randomized clinical trial, comparing these agents with a placebo.

## Methods

### Study design, patients and treatments

This post hoc exploratory analysis used the data of patients with moderately to severely active RA who participated in the randomized, double-blind, placebo-controlled and active-controlled phase III study (clinicaltrials.gov NCT02760407, CREDO-2) and received OKZ 64 mg once every 2 weeks (q2w) or 4 weeks (q4w), ADA 40 mg q2w or placebo for 24 weeks of the study treatment period [13]. All patients who entered the trial had responded inadequately to methotrexate (MTX), had no bDMARDs or tsDMARDs exposure before OKZ/ADA/placebo initiation and received background MTX therapy. The CREDO-2 study included 1648 subjects with RA, and most patients completed 24 weeks of treatment: 421 (90.7%) in the OKZ q2w arm, 437 (91.2%) in the OKZ q4w arm, 413 (89.4%) in the ADA arm, and 208 (85.6%) in the placebo arm. The study protocol was reviewed and approved by the responsible local institutional review boards or ethics committees. This post hoc analysis of the CREDO-2 clinical trial data addressed the impact of the comorbidity burden on the treatment outcomes of two bDMARDs with different mechanisms of action and placebo.

### Assessments and endpoints

To assess the effects of comorbid conditions on outcomes, patients were divided into two groups on the basis of the modified Charlson Comorbidity Index (mCCI) at baseline: those having only RA with no comorbid conditions (NCC; mCCI = 1; i.e., RA only) vs. those having RA and comorbid conditions (CC; mCCI ≥ 2; i.e., RA plus ≥ 1 comorbidity). The Charlson Comorbidity Index (CCI) [14] is a weighted score of comorbid conditions that was validated for the prediction of mortality, hospital stay, functional disability, and healthcare utilization. It is one of the most widely used comorbidity indices [15, 16]. This index was previously used to assess the impact of comorbidities on RA treatment efficacy and persistence [17–21]. The CCI includes 19 conditions: myocardial infarction, congestive cardiac failure, peripheral artery disease, cerebrovascular disease, chronic pulmonary disease, peptic ulcer disease, liver and renal diseases, diabetes, etc. The mCCI was calculated as the sum of investigator-reported comorbidities based on the CREDO-2 baseline data (see Supplementary material, Table S1). Since the CCI includes connective tissue diseases, RA was coded as ‘1’ for all the patients. Age points in the CCI were excluded in mCCI since the aim of the analysis was the assessment of comorbidity burden rather than mortality prediction [14, 20].

The key outcomes at Week 24 (W24) were the proportions of patients who achieved a CDAI ≤ 10 (low disease activity, LDA) and the proportions of patients who achieved CDAI ≤ 2.8 (remission, REM). American College of Rheumatology 50 (ACR50) response at Week 12 (W12) and W24 and the proportions of patients who achieved (SDAI) ≤ 3.3 (SDAI REM) at W24 were also analyzed. Among these composite measures, the CDAI is CRP independent, so the CDAI-related outcomes (CDAI LDA and CDAI REM) at W24 were the key outcome measures, whereas other composite outcomes include CRP and were the co-measures. CDAI-REM is an ACR-EULAR ratified measure of remission [22, 23]. The endpoints were chosen based on the EULAR recommendations for the management of rheumatoid arthritis with synthetic and biological DMARDs to reflect the treat-to-target approach, which includes at least a 50% improvement in disease activity within 3 months and remission or low disease activity by 6 months [24]. Safety outcomes included treatment-emergent adverse events (AEs) and serious adverse events (SAEs).

### Statistical analysis

Demographics and other baseline characteristics are presented via descriptive statistics by patient comorbidity status (NCC and CC). The comparison of outcomes between the NCC and CC groups was performed using chi-square tests for categorical variables and independent-samples t-tests for continuous variables. Treatment outcomes at W12 and W24 were assessed using multivariable logistic regression models for binary key and other outcomes (composite measures). To estimate the odds ratios (OR) with 95% confidence intervals (CIs), two logistic regression models were used: a crude model (Model 1) and an adjusted model (Model 2), which included age, sex, baseline value of the respective outcome variable, disease duration, and prior MTX exposure. The baseline value for the CDAI ≤ 10 and CDAI ≤ 2.8 response rates was the baseline CDAI score, for the SDAI ≤ 3.3 response rate it was the baseline SDAI score, and for the ACR50 response rate it was the baseline DAS28(CRP) score. Interaction terms between comorbidity status (NCC vs. CC) and treatment group (placebo as reference) were included to test for effect modification. P-values for interaction terms were used to identify whether treatment effects differed significantly by comorbidity strata. This analysis was performed in the intent-to-treat population of the CREDO-2 study. The data collected after initiation of rescue medication for non-responders were retained in the analysis. A non-responder imputation (NRI) approach was used for patients with missing outcome data due to early discontinuation prior to W12 or W24. For different outcome measures, 12.1–30.8%, 8.9–22.6% and 12.2–22.7% of the data were missing in the placebo, OKZ and ADA groups, respectively, largely due to an early withdrawal (Table S8). Descriptive statistics was used to summarize AEs, SAEs data, and the distribution of baseline comorbidities; the frequencies were compared between treatment groups using chi-square tests or Fisher’s exact tests. All the statistical analyses were performed in R 4.4.1.

## Results

### Patient baseline characteristics

A total of 1645 patients with RA were included in this post hoc analysis of the CREDO-2 trial (NCT02760407). Among them, 463 patients were treated with OKZ 64 mg q2w, 477 patients were treated with OKZ 64 mg q4w, 243 patients received placebo, and 462 patients were treated with ADA 40 mg q2w. Among the OKZ-treated patients, in the q2w arm, 348 (75.2%) patients were in the NCC and 115 (24.8%) in the CC groups; in the q4w arm, 353 (74.0%) with NCC and 124 (26.0%) with CC. Among the ADA-treated patients, 352 (76.2%) had no comorbidities, and 110 (23.8%) had comorbidities. In the placebo-treated group, 185 (76.1%) patients were in the NCC, and 58 (23.9%) in the CC groups.

The patients’ demographic and baseline characteristics are presented in the Table 1. There were expected differences in baseline characteristics, namely, age and body mass index (BMI), between the CC and the NCC groups of patients, as detailed in the Table 1.

**Table 1:** Demographic and other baseline characteristics

Variable	OKZ q2w		OKZ q4w		ADA		Placebo	
	NCC *N* = 348	CC *N* = 115	NCC *N* = 353	CC *N* = 124	NCC *N* = 352	CC *N* = 110	NCC *N* = 185	CC *N* = 58
Gender								
Male, n (%)	78 (22.4)	33 (28.7)	70 (19.8)	32 (25.8)	70 (19.9)	29 (26.4)	35 (18.9)	18 (31.0)
Female, n (%)	270 (77.6)	82 (71.3)	283 (80.2)	92 (74.2)	282 (80.1)	81 (73.6)	150 (81.1)	40 (69.0)
p-value	0.171		0.163		0.148		0.051	
Age, years, Mean ± SD	50.9 ± 11.9	60.3 ± 9.0	51.8 ± 12.2	59.3 ± 10.0	52.9 ± 12.6	58.6 ± 10.2	53.2 ± 11.9	59.7 ± 10.1
p-value	**< 0.001**		**< 0.001**		**< 0.001**		**< 0.001**	
BMI, kg/m^2^, Mean ± SD	28.3 ± 6.0	29.6 ± 6.3	27.7 ± 5.7	31.5 ± 7.0	28.1 ± 6.4	29.9 ± 5.4	28.0 ± 6.2	30.5 ± 7.6
p-value	**0.032**		**< 0.001**		**0.004**		**0.027**	
Race								
White, n (%)	279 (80.2)	103 (89.6)	291 (82.4)	113 (91.1)	290 (82.4)	95 (86.4)	152 (82.2)	51 (87.9)
Black, n (%)	16 (4.6)	4 (3.5)	9 (2.5)	6 (4.8)	18 (5.1)	5 (4.5)	9 (4.9)	2 (3.4)
Asian, n (%)	8 (2.3)	2 (1.7)	6 (1.7)	0	2 (0.6)	2 (1.8)	5 (2.7)	0
Other, n (%)	45 (12.9)	6 (5.2)	47 (13.3)	5 (4.0)	42 (11.9)	8 (7.3)	19 (10.3)	5 (8.6)
p-value	0.098		**0.004**		0.292		0.734	
Duration of RA, years, Mean ± SD	7.1 ± 7.1	8.4 ± 8.7	7.4 ± 6.9	7.5 ± 7.3	7.6 ± 7.6	6.7 ± 7.1	7.4 ± 7.8	5.4 ± 4.3
p-value	0.676		0.920		0.268		**0.016**	
Smoking status								
Never smoking, n (%)	236 (67.8)	64 (55.7)	246 (69.7)	68 (54.8)	235 (66.8)	67 (60.9)	118 (63.8)	32 (55.2)
Previous smoking (former), n (%)	47 (13.5)	20 (17.4)	49 (13.9)	28 (22.6)	47 (13.4)	27 (24.5)	31 (16.8)	13 (22.4)
Current smoking, n (%)	64 (18.4)	28 (24.3)	55 (15.6)	28 (22.6)	69 (19.6)	16 (14.5)	36 (19.5)	13 (22.4)
p-value	0.137		**0.006**		**0.017**		0.472	
Alcohol status								
Never consumption, n (%)	239 (68.7)	61 (53.0)	251 (71.1)	74 (59.7)	237 (67.3)	72 (65.5)	125 (67.6)	35 (60.3)
Previous consumption (former), n (%)	18 (5.2)	6 (5.2)	16 (4.5)	6 (4.8)	12 (3.4)	4 (3.6)	8 (4.3)	3 (5.2)
Current consumption, n (%)	90 (25.9)	45 (39.1)	83 (23.5)	44 (35.5)	103 (29.3)	34 (30.9)	52 (28.1)	20 (34.5)
p-value	**0.019**		**0.038**		0.936		0.599	
Duration of prior MTX use, weeks, Mean ± SD	179.9 ± 211.5	218.2 ± 275.3	193.8 ± 208.4	181.6 ± 235.2	204.3 ± 244.9	195.7 ± 229.5	203.2 ± 242.6	170.6 ± 189.1
p-value	0.708		0.156		0.739		0.289	
Dose of MTX, mg/week, Mean ± SD	17.1 ± 4.0	16.9 ± 4.3	17.0 ± 4.0	17.8 ± 4.0	17.2 ± 4.0	17.7 ± 4.1	17.0 ± 3.7	17.5 ± 4.7
p-value	0.640		0.083		0.225		0.475	
Glucocorticoids use, n (%)	225 (65%)	62 (54%)	220 (62%)	65 (52%)	210 (59.7)	56 (50.9)	120 (64.9)	35 (60.3)
p-value	0.040		0.054		0.131		0.640	
Dose of prednisone equivalent use, mg/day, Mean ± SD	6.4 ± 3.2	7.6 ± 6.6	6.9 ± 4.5	5.8 ± 2.2	7.6 ± 6.7	7.3 ± 5.7	10.6 ± 36.3	8.9 ± 16.0
p-value	0.238		0.064		0.707		0.693	
CDAI, Mean ± SD	39.8 ± 11.7	39.8 ± 10.5	39.4 ± 11.4	40.9 ± 12.2	39.0 ± 11.6	40.1 ± 11.8	39.1 ± 12.2	39.6 ± 9.3
p-value	0.959		0.294		0.458		0.750	
SDAI, Mean ± SD	41.8 ± 11.9	41.6 ± 11.0	41.4 ± 11.9	42.5 ± 12.5	41.0 ± 12.1	41.6 ± 11.9	40.7 ± 12.5	41.4 ± 9.7
p-value	0.854		0.355		0.644		0.700	
DAS28(CRP), Mean ± SD	5.9 ± 0.8	5.9 ± 0.8	5.8 ± 0.8	5.9 ± 0.9	5.9 ± 0.9	5.9 ± 0.8	5.8 ± 0.9	6.0 ± 0.7
p-value	0.747		0.238		0.966		0.171	
HAQ Disability Index, Mean ± SD	1.71 ± 0.60	1.79 ± 0.52	1.67 ± 0.60	1.77 ± 0.58	1.69 ± 0.57	1.79 ± 0.55	1.69 ± 0.61	1.77 ± 0.64
p-value	0.397		0.158		0.119		0.418	
CRP, mg/L, Mean ± SD	19.6 ± 21.4	17.5 ± 20.3	19.1 ± 19.2	16.5 ± 17.0	19.6 ± 19.6	15.6 ± 14.0	16.8 ± 17.9	18.1 ± 14.5
p-value	0.287		0.318		**0.021**		0.575	
Pain by VAS, mm, Mean ± SD	68.4 ± 21.2	68.6 ± 18.7	67.1 ± 20.6	67.0 ± 22.1	66.1 ± 21.5	69.3 ± 20.4	66.5 ± 20.3	66.6 ± 20.3
p-value	0.803		0.850		0.233		0.843	
PhGA, mm, Mean ± SD	65.6 ± 16.5	68.0 ± 15.0	66.7 ± 15.7	67.1 ± 16.5	64.5 ± 17.5	67.5 ± 15.3	64.4 ± 16.4	68.8 ± 15.4
p-value	0.284		0.580		0.138		0.111	
PtGA, mm, Mean ± SD	66.8 ± 21.1	69.5 ± 17.1	65.9 ± 20.7	69.3 ± 21.1	66.3 ± 21.1	68.5 ± 20.4	67.5 ± 19.1	67.1 ± 20.8
p-value	0.470		0.090		0.394		0.957	
TJC28, Mean ± SD	15.4 ± 6.5	15.2 ± 6.4	14.7 ± 6.2	15.5 ± 6.3	14.6 ± 6.0	15.4 ± 6.2	13.9 ± 6.2	15.3 ± 5.5
p-value	0.802		0.142		0.203		0.158	
TJC68, Mean ± SD	23.9 ± 12.5	24.0 ± 12.5	23.1 ± 12.4	25.2 ± 14.2	23.2 ± 12.3	26.2 ± 13.3	21.7 ± 12.2	23.4 ± 11.8
p-value	0.892		0.208		0.038		0.465	
SJC28, Mean ± SD	15.4 ± 6.5	15.2 ± 6.4	14.7 ± 6.2	15.5 ± 6.3	11.3 ± 4.9	10.8 ± 5.1	11.1 ± 4.9	10.7 ± 4.7
p-value	0.641		0.878		0.264		0.442	
SJC66, Mean ± SD	14.7 ± 7.2	14.3 ± 7.3	15.3 ± 8.5	15.8 ± 9.6	15.4 ± 7.7	15.3 ± 8.7	15.1 ± 8.5	14.0 ± 8.3
p-value	0.724		0.960		0.852		0.266	

*Abbreviations*: *ADA* adalimumab, *BMI* body mass index, *CC* comorbid conditions, *CDAI* clinical disease activity index, *CRP* C-reactive protein, *DAS28* disease activity score 28-joint count, *HAQ* health assessment questionnaire, *MTX* methotrexate, *N* number of patients in the arm, *NCC* no comorbid conditions, *OKZ* olokizumab, *PhGA* physician global assessment, *PtGA* patient global assessment, *q2w* once every 2 weeks, *q4w* once every 4 weeks, *RA* rheumatoid arthritis, *SD* standard deviation, *SDAI* simplified disease activity index, *SJC* swollen joint count, *TJC* tender joint count, *VAS* visual analog scale

Comparison between groups was performed using chi-square tests for categorical variables and independent-samples t-tests for continuous variables. Bold font indicates statistical significance (*p*-value < 0.050)

There were some incidental significant differences between the treatment groups in the frequency of baseline comorbidities, with a trend towards a higher number of patients with CC in the OKZ-treated groups and a lower number of patients with CC in the placebo group. The most common comorbidities in patients who had at least one comorbidity were diabetes without chronic complications (33.0% in the OKZ q2w arm, 38.7% in the OKZ q4w arm, 37.3% in the ADA arm, and 50.0% in the placebo arm), chronic pulmonary disease (30.4% in the OKZ q2w arm, 27.4% in the OKZ q4w arm, 36.4% in the ADA arm, and 33.0% in the placebo arm), and mild liver disease (14.8% in the OKZ q2w arm, 21.8% in the OKZ q4w arm, 14.5% in the ADA arm, and 5.0% in the placebo arm). Acquired immunodeficiency syndrome, leukemia, lymphoma, and solid-tumor cancers, which constitute variables in the mCCI, were not present at baseline in the CREDO-2 patients (Table 2). There was a numerically higher proportion of patients with cardiovascular diseases at baseline, including previous myocardial infarction, heart failure, and cerebrovascular disease, in the OKZ q2w arm than in the OKZ q4w arm (Table 2). The overwhelming majority of the patients in all treatment groups (see Supplementary material, Table S2) had one comorbid condition (CCI = 2). The relatively low number of multimorbid patients was due to the study’s exclusion criteria: patients with uncontrolled diabetes or hypertension, severe heart failure, etc., were not included in the clinical trial.

**Table 2:** Distribution of comorbidities included in the mCCI

Comorbidity	OKZ q2w		OKZ q4w		ADA		Placebo		*p*-value
	NCC (*N* = 348) *n* (%)	CC (*N* = 115) *n* (%)	NCC (*N* = 353) *n* (%)	CC (*N* = 124) *n* (%)	NCC (*N* = 352) *n* (%)	CC (*N* = 110) *n* (%)	NCC (*N* = 185) *n* (%)	CC (*N* = 58) *n* (%)	
Diabetes without chronic complications	0	38 (33.0)	0	48 (38.7)	0	41 (37.3)	0	29 (50.0)	0.194
Chronic pulmonary disease	0	35 (30.4)	0	34 (27.4)	0	40 (36.4)	0	19 (33.0)	0.535
Liver disease, mild	0	17 (14.8)	0	27 (21.8)	0	16 (14.5)	0	3 (5.0)	**0.030**
Myocardial infarction	0	14 (12.2)	0	10 (8.1)	0	8 (7.3)	0	4 (7.0)	0.438
Congestive heart failure	0	15 (13.0)	0	7 (5.6)	0	5 (4.5)	0	0	**0.005**
Peptic ulcer disease	0	8 (7.0)	0	12 (9.7)	0	12 (10.9)	0	4 (7.0)	0.706
Cerebrovascular disease	0	13 (11.3)	0	3 (2.4)	0	11 (10.0)	0	3 (5.0)	**0.025**
Peripheral vascular disease	0	9 (7.8)	0	6 (4.8)	0	6 (5.5)	0	2 (3.0)	0.694
Renal disease, mild to moderate	0	2 (1.7)	0	5 (4.0)	0	2 (1.8)	0	2 (3.0)	0.675
Diabetes with chronic complications	0	3 (2.6)	0	2 (1.6)	0	3 (2.7)	0	3 (5.0)	0.823
Hemiplegia or paraplegia	0	0	0	1 (0.8)	0	0	0	0	> 0.999
Liver disease, moderate to severe	0	1 (0.9)	0	0	0	0	0	0	0.695
Renal disease, severe	0	0	0	0	0	0	0	1 (2.0)	0.143

*Abbreviations*: *ADA* adalimumab, *CC* comorbid conditions, *OKZ* olokizumab, *N* number of patients in the arm, *n* the number of patients, *NCC* no comorbid conditions, *q2w* every 2 weeks, *q4w* every 4 weeks

Comparisons between groups were performed using the chi-square test or Fisher’s exact test

### Impact of comorbidities on individual efficacy outcomes

**Fig. 1:**
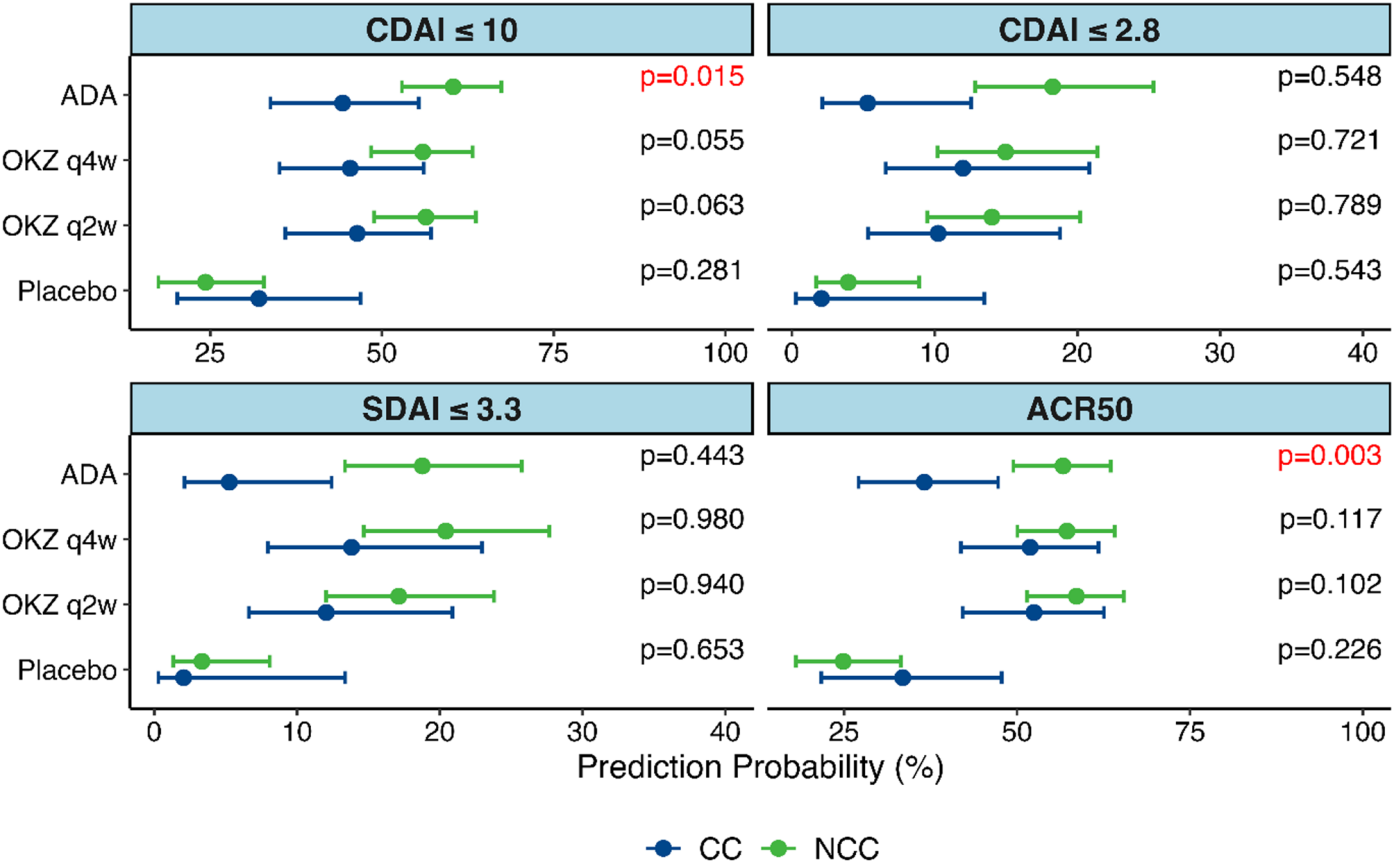
Predicted Probability with 95% CI from logistic regression for the key and other outcomes. Model 2. Week 24. Abbreviations: ACR 50, 50% improvement in the American College of Rheumatology criteria; ADA, adalimumab; CC, comorbid conditions; CDAI, Clinical Disease Activity Index; NCC, no comorbid conditions; OKZ, olokizumab; q2w – once every 2 weeks; q4w – once every 4 weeks, SDAI, Simplified Disease Activity Index. Note: *p*-value for interaction terms in logistic regression is presented

At W24, the adjusted analysis (Model 2) of the key endpoints revealed statistically significant differences in predicted probability only in the ADA-treatment arm between NCC and CC groups for CDAI ≤ 10, with a higher response rate observed in the NCC group. There were no differences for CDAI ≤ 2.8 in all the treatment arms; however, the number of patients with CDAI REM in each of the analyzed groups was small. The number of patients who achieved each endpoint at W12 and W24 is listed in the Table S3. In addition, a difference was observed for ACR50 outcome in the ADA-treated patients, with a significantly higher number of responders in the NCC group (Fig. 1, Supplementary material, Table S6). The differences between NCC and CC groups in predicted probability for CDAI ≤ 10 and ACR50 outcomes were observed for both models analyzed in the ADA-treatment arm (Supplementary material, Table S6). This finding was also confirmed by OR interaction analysis for the two outcomes in both models (Supplementary material, Table S6). In contrast to ADA, in the OKZ-treatment arms and in the placebo arm no differences in predicted probability between NCC and CC groups were found (Fig. 1, Supplementary material, Tables S6, S7).

**Fig. 2:**
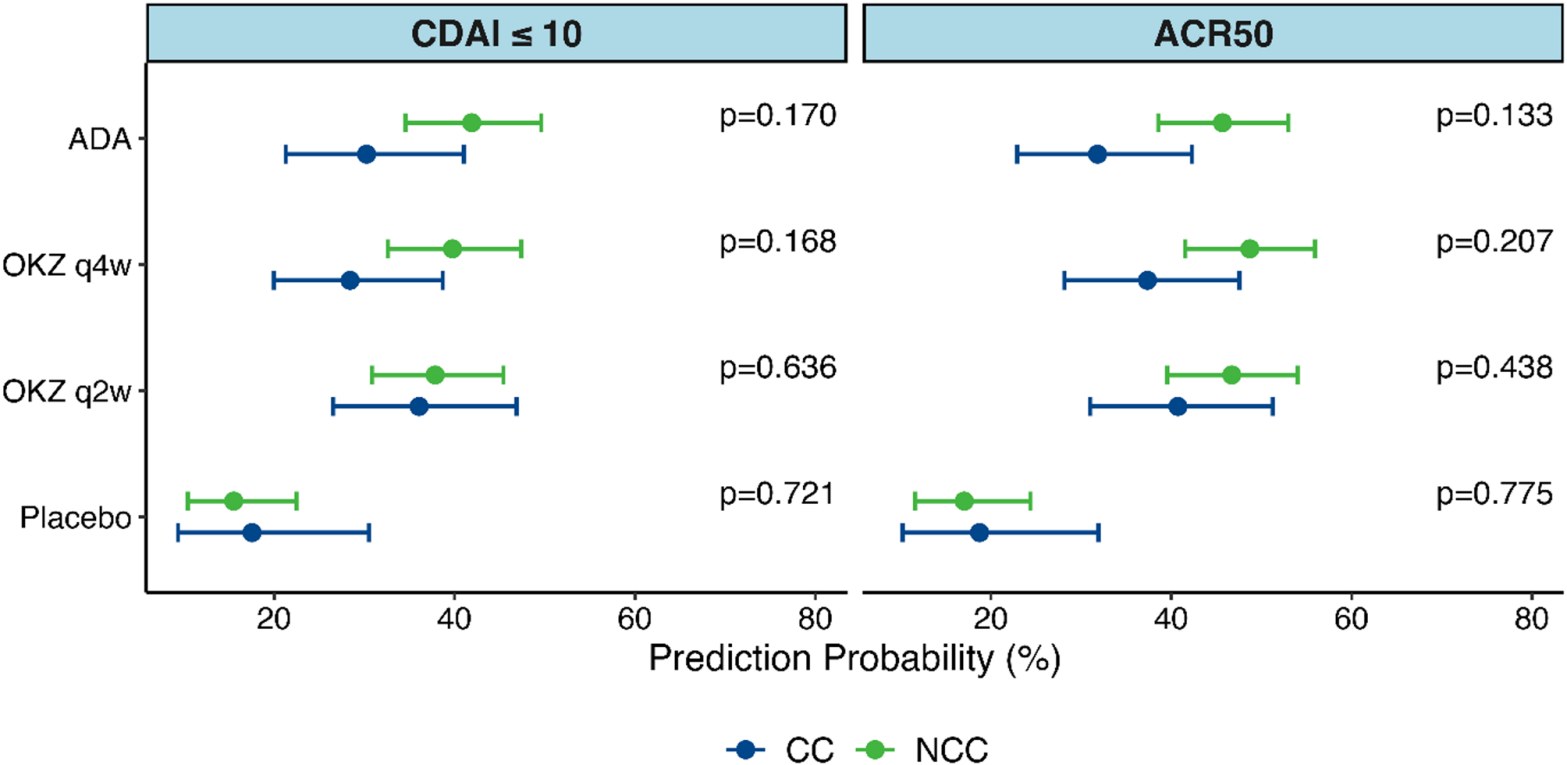
Predicted Probability for the key and other outcomes. Model 2. Week 12. Abbreviations: ACR 50, 50% improvement in the American College of Rheumatology criteria; ADA, adalimumab; CC, comorbid conditions; CDAI, Clinical Disease Activity Index; NCC, no comorbid conditions; OKZ, olokizumab; q2w – once every 2 weeks; q4w – once every 4 weeks; Note: p-value for interaction terms in logistic regression is presented

The adjusted analysis (Model 2) revealed no significant differences in predicted probability and odds ratios (OR) for achieving the endpoints of the exploratory analysis by W12 in all the treatment arms (Fig. 2, Supplementary material, Table S4, Table S5).

It is worthy of note that at Week 12 the values of predicted probabilities regarding CDAI-LDA for the CC and NCC groups were similar for the ADA-treatment arm and both OKZ-treatment arms, with higher probabilities compared to the placebo group (Fig. 2).

### Safety outcomes

**Fig. 3:**
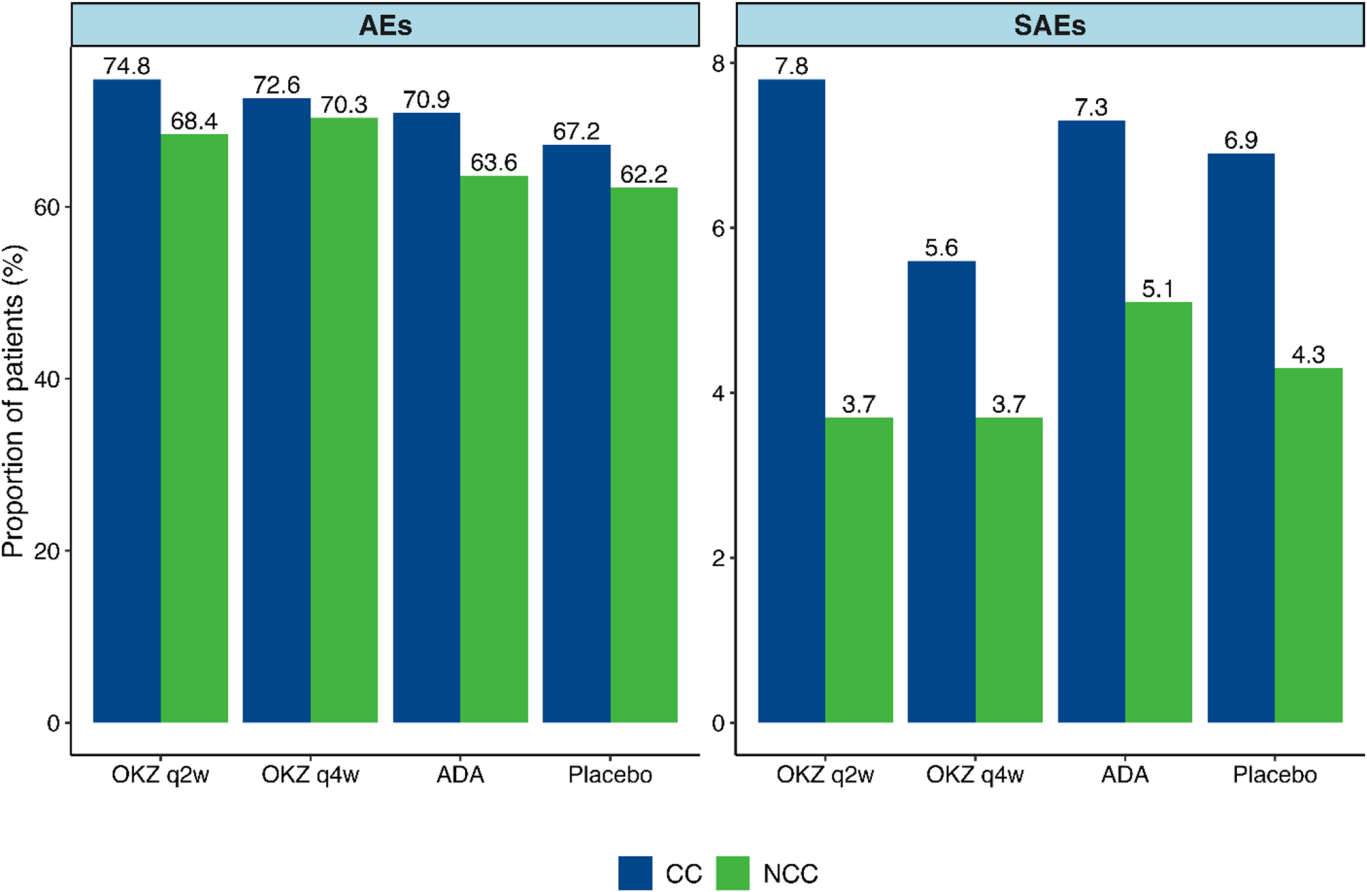
Proportion of patients with at least one AE/SAE during the study. Abbreviations: ADA, adalimumab; AE, adverse event; CC, comorbid conditions; mCCI, modified Charlson comorbidity index; NCC, no comorbid conditions; OKZ, olokizumab; q2w, once every 2 weeks; q4w, once every 4 weeks; SAE, serious adverse event

Similar proportions of patients experienced AEs or SAEs between comorbidity subgroups in OKZ q2w and q4w-, placebo-, and ADA-treated patients, with a trend toward higher rates in patients with CC, as expected. There was no correlation between the NCC or CC groups and the occurrence of certain AEs. The incidence rates of AEs and SAEs in the placebo, ADA, and both OKZ treatment groups are presented in the Fig. 3.

## Discussion

This post hoc analysis aimed to assess the impact of comorbidities on treatment outcomes in patients treated with OKZ or ADA compared to placebo in this phase III randomized active-controlled clinical trial. All patients in the CREDO-2 study had active moderate to severe RA, had previously failed MTX treatment, and had OKZ or ADA as the first bDMARD. Moreover, CREDO-2 is the first trial that compared a monoclonal antibody directed against the IL-6 pathway in combination with MTX to an anti-TNF plus MTX. Previous trials comparing tocilizumab or sarilumab against adalimumab used these agents in monotherapy and not in combination with MTX [25, 26].

The comorbidities observed in the present study are common in patients with RA [1]. The most common CCI comorbidities were diabetes mellitus and chronic pulmonary disease. Notably, owing to the strict eligibility criteria for the clinical trial (exclusion of patients with malignancies, uncompensated congestive heart failure, history or presence of any concurrent severe and/or uncontrolled medical condition, etc.), the representation of comorbidities differs from that of the general population of patients with RA, as represented in registries. For example, in the CORRONA RA registry study of tocilizumab (TCZ) [20], solid-tumor cancer was reported in 21.0% of patients, whereas no patient had solid-tumor cancer in the CREDO-2 study sample at inclusion in the study.

The general results of our study are in line with data from previous reports, which demonstrate the influence of the burden of comorbidities on bDMARDs efficacy. Indeed, several studies have reported the relationship between the comorbidity burden and a reduced probability of achieving a clinical response in TNFi-treated RA patients. In a recent analysis of the BIOBADASER cohort, a higher CCI was associated with a higher DAS28 in TNFi-treated RA patients, but no differences in remission rates were found [27]. In the study of Biggioggero et al. [28], an increase in the Rheumatic Disease Comorbidity Index (RDCI) was shown to be a predictor of a lower likelihood of achieving a 1-year EULAR good–moderate response (*p* = 0.02) in TNFi-treated patients. In the analysis of the RADIUS-2 cohort of RA patients receiving etanercept, fewer comorbidities were associated with greater improvements in HAQ-DI score and CDAI-LDA response (*p* = 0.001), and each additional comorbidity decreased the odds ratio of achieving a CDAI-LDA score by 18% [29]. There is evidence that patients who discontinue TNFi have higher CCI scores than those who do not [19, 30, 31], which may be due to a lower treatment response in patients with higher comorbidity scores. Burmester et al. [32] reported that ADA-treated patients who had one or no comorbidities were more likely to achieve RA remission than patients with two or more comorbidities were (OR 0.86, 95% CI 0.780–0.932; *p* = 0.0005).

Similar results were obtained in our analysis for the ADA group, where several efficacy outcomes, including CDAI-LDA achievement, were influenced by comorbidities by 6 months of treatment.

At 12 weeks, the difference between NCC and CC was not observed for ADA, but this is likely due to the fact that the profound efficacy responses evaluated here, such as ACR 50 (or LDA), do not peak at 12 weeks but rather later and, therefore, the difference could only be observed at the time of maximum efficacy of these endpoints [33].

Different results have been obtained to date for IL-6 receptor inhibitors. Stajszczyk et al. [34] reported that when TCZ was used as a first-line biologic treatment in patients with moderate-to-severe RA, RDCI did not correlate with the proportion of patients who achieved DAS28 LDA at 3 and 6 months or DAS28 REM at 6 months of treatment. Pappas et al. [20] reported that the effectiveness of TCZ in RA patients from the CORRONA registry did not differ by comorbidity status. Patients in both comorbidity statuses in this study [20] improved from baseline in the CDAI and HAQ at 6 months, with no significant differences between the high-mCCI and low-mCCI groups.

In the current analysis, in both the CC and NCC cohorts of OKZ-treated patients, no difference was observed across outcomes for both q4w and q2w treatment regimens. Our analysis revealed that the presence of comorbid conditions did not diminish the treatment efficacy of OKZ; both comorbid and non-comorbid patient groups in the CREDO-2 study achieved similar improvements in CDAI LDA and CDAI REM rates at 6 months. As expected, no differences in LDA or REM were observed for the placebo arm: in this treatment arm, significantly fewer patients reached these endpoints over time compared with patients receiving active therapy.

Notably, none of the studies discussed above included both an anti-TNF agent and an agent that inhibits the IL-6 pathway in the same study and same trial design. Thus, the strength of the present study is the direct assessment of comorbidity data in a head-to-head trial comparing the efficacy of OKZ with that of ADA and placebo, all in combination with MTX.

Compared with ADA, treatment with OKZ resulted in similar rates of positive clinical outcomes in RA patients with and without comorbidities. The background of these differences is unknown, and any rationalization must be considered speculative. Nevertheless, a reasonable explanation is related to the effects of comorbidities on outcomes, as response rates decrease [35] with increasing CCI scores [36]. Since IL-6 blockade has positive effects on glycosylated hemoglobin levels compared with TNF inhibition, and since it also decreases NT-proBNP levels, increases the left ventricular ejection fraction, and decreases the left ventricular mass index in patients with RA [37–40], it is conceivable that the overall comorbidity status of patients may not be affected by ADA, whereas IL-6 blockade by OKZ may improve it. Under these circumstances, ADA responses are affected by the comorbid situation, which is in line with previous observations [19, 28, 29, 41], whereas IL-6 blockade may reduce the negative effects of comorbid conditions on outcomes. Of course, such an assumption will have to be investigated prospectively by evaluating mCCI over the course of a trial.

Our study has several limitations. First, this was an exploratory post hoc analysis of a clinical trial; however, the trial itself was randomized, prospective, and masked, and comorbidities were assessed at baseline without prejudice or knowledge of the treatment arms. Second, the data were obtained in the RCT rather than in the real world or from registries, so patients with some severe/unstable comorbidities were excluded. Third, the sample sizes of OKZ and ADA versus placebo were not equal since randomization occurred at a 2:2:2:1 (1 for placebo) ratio. Fourth, the influence of some individual comorbidities was not analyzed because of their low prevalence in the CREDO-2 population, so we cannot provide data on their influence on treatment efficacy. The limited prevalence of comorbidities also prevented us from analyzing the stratified mCCI (mCCI = 2, mCCI = 3, mCCI = 4, etc.). Fifth, the observation period was limited by the study duration. Sixth, the severity of the individual comorbidities was not assessed. Seventh, response rates for the most stringent endpoints, CDAI- and SDAI-remission, were low. These low event rates reduced the statistical power, increasing the risk of failing to detect a true treatment effect. Thus, the data presented, while compelling, cannot be regarded as definitive and has to be confirmed in future analyses. However, and this is a major strength of our study, we did not have to perform indirect comparisons but could use data from a head-to-head trial of OKZ vs. ADA and, in addition, had two OKZ arms available whose results were essentially cross-confirmatory.

## Conclusions

In conclusion, this post hoc analysis of the phase III CREDO-2 study suggests that CC (or the presence of at least one comorbid condition in the mCCI, including such common disorders as chronic pulmonary diseases and cardiovascular diseases) does not affect OKZ q2w and OKZ q4w treatment results in RA patients at both 3 and 6 months of treatment. In contrast, comorbidities reduce CDAI LDA and ACR50 outcomes upon ADA treatment at 6 months, highlighting the reduced responsiveness to ADA among patients with comorbidities. There was no influence of the mCCI on safety outcomes or placebo group results. OKZ efficacy and safety in patients with RA with comorbidities were consistent with the overall RA phase III study population and other studies.

## Supplementary Information

Supplementary Material 1.

## Data Availability

This is a post hoc analysis. All the relevant information was provided in the publication that describes the main study results (Smolen JS, Feist E, Fatenejad S, et al. Olokizumab versus Placebo or Adalimumab in Rheumatoid Arthritis. N Engl J Med. 2022;387(8):715-726) at https://www.nejm.org/doi/10.1056/NEJMoa2201302. Upon a reasonable request to bukhanova@rpharm.ru, R-Pharm will provide access to individual deidentified participant data depending upon the nature of the request, the merit of the proposed research, the availability of the data, and its intended use.

## Abbreviations

ACR: American College of Rheumatology
ACR50: American College of Rheumatology 50% Response Criteria
ADA: Adalimumab
AE: Adverse Event
bDMARD: Biologic Disease-Modifying Antirheumatic Drug
BMI: Body Mass Index
CC: Comorbid conditions
CCI: Charlson Comorbidity Index
CDAI: Clinical Disease Activity Index
csDMARD: Conventional Synthetic Disease-Modifying Antirheumatic Drug
CI: Confidence Interval
CRP: C-Reactive Protein
DAS28: Disease Activity Score 28-Joint Count
DMARD: Disease-Modifying Antirheumatic Drug
HAQ: Health Assessment Questionnaire
IL-6: Interleukin-6
LDA: Low Disease Activity
mAb: Monoclonal Antibody
mCCI: Modified Charlson Comorbidity Index
MTX: Methotrexate
NCC: No Comorbid Conditions
NRI: Non-Responder Imputation
OKZ: Olokizumab
OR: Odds Ratio
PhGA: Physician Global Assessment
PtGA: Patient Global Assessment
q2w: Once Every 2 Weeks
q4w: Once Every 4 Weeks
RA: Rheumatoid Arthritis
REM: Remission
SAE: Serious Adverse Event
SD: Standard Deviation
SDAI: Simplified Disease Activity Index
SJC: Swollen Joint Count
TCZ: Tocilizumab
TJC: Tender Joint Count
TNF: Tumour Necrosis Factor
TNFi: Tumour Necrosis Factor Inhibitor
tsDMARD: Targeted Synthetic Disease-Modifying Antirheumatic Drug
VAS: Visual Analog Scale
W: Week
