# Supplementary material for: The impact of comorbidities on the efficacy of IL-6 inhibitor olokizumab compared to adalimumab in a randomized controlled trial
Source: Arthritis Res Ther. 2025 Nov 14;27:213. doi: 10.1186/s13075-025-03682-w (PMC12619491; doi:10.1186/s13075-025-03682-w)
Supplement: Supplementary file 1 — Supplementary Material 1. [file 13075_2025_3682_MOESM1_ESM.docx]

**SUPPLEMENTARY MATERIAL**

**Abbreviations**

| ACR | American College of Rheumatology |
| --- | --- |
| ACR20 | American College of Rheumatology 20% Response Criteria |
| ACR50 | American College of Rheumatology 50% Response Criteria |
| ADA | Adalimumab |
| AE | Adverse Event |
| CC | Comorbid Conditions |
| CCI | Charlson Comorbidity Index |
| CDAI | Clinical Disease Activity Index |
| CI | Confidence Interval |
| CRP | C-Reactive Protein |
| DAS28 | Disease Activity Score 28-Joint Count |
| mCCI | Modified Charlson Comorbidity Index |
| MedDRA | Medical Dictionary for Regulatory Activities |
| NCC | No Comorbid Conditions |
| OKZ | Olokizumab |
| OR | Odds Ratio |
| q2w | Once Every 2 Weeks |
| q4w | Once Every 4 Weeks |
| SDAI | Simplified Disease Activity Index |

Table S1. mCCI Scoring System based on study CREDO2 results

| **Condition Number** | **Condition Description** | **Points** | **MedDRA Term** | |
| --- | --- | --- | --- | --- |
|  |  |  | **Concomitant condition** | **Medical History** |
| 1 | Myocardial infarction | 1 | Myocardial fibrosis | Acute myocardial infarction, Myocardial infarction |
| 2 | Congestive heart failure | 1 | Cardiac failure chronic, Cardiac failure congestive, Cardiac hypertrophy,  Cardiomyopathy, Chronic left ventricular failure,  Congestive cardiomyopathy, Diastolic dysfunction,  Hypertensive cardiomyopathy, Ischaemic cardiomyopathy, Metabolic cardiomyopathy | - |
| 3 | Peripheral vascular disease | 1 | Arteriosclerosis, Aortic aneurysm, Aortic arteriosclerosis, Aortic dilatation, Aortic disorder, Aortic stenosis, Peripheral vascular disorder, Aortic dissection | - |
| 4 | Cerebrovascular disease | 1 | Carotid artery stenosis, Cerebral artery stenosis, Cerebral ischaemia, Cerebrovascular insufficiency, Transient ischaemic attack, Cerebrovascular disorder, Vertebral artery stenosis | Cerebellar stroke, Cerebral haemorrhage, Cerebral ischaemia, Cerebrovascular accident, Ischaemic stroke, Transient ischaemic attack |
| 5 | Dementia | 1 | - | - |
| 6 | Chronic pulmonary disease | 1 | Asthma, Bronchiectasis, Bronchitis chronic, Chronic obstructive pulmonary disease, Emphysema, Pneumoconiosis | - |
| 8 | Peptic ulcer disease | 1 | Duodenal ulcer; Gastric ulcer; Peptic ulcer; Gastroduodenal ulcer; Gastrointestinal ulcer | Duodenal ulcer; Gastric ulcer; Peptic ulcer; Gastroduodenal ulcer; Gastrointestinal ulcer, Gastric ulcer perforation |
| 9 | Liver disease, mild | 1 | Hepatic steatosis, Hepatitis, Non-alcoholic steatohepatitis, Steatohepatitis, Liver disorder, Nonalcoholic fatty liver disease, Drug-induced liver injury | - |
| 10 | Diabetes without chronic complications | 1 | Diabetes mellitus, Type 2 diabetes mellitus, Type 1 diabetes mellitus, Insulin resistant diabetes, Steroid diabetes | - |
| 11 | Renal disease, mild to moderate | 1 | Chronic kidney disease | - |
| 12 | Diabetes with chronic complications | 2 | Diabetic nephropathy, Diabetic neuropathy | - |
| 13 | Hemiplegia or paraplegia | 2 | Cerebral palsy | - |
| 14 | Any malignancy | 2 | Brain neoplasm malignant | - |
| 15 | Liver disease, moderate to severe | 3 | Portal hypertensive gastropathy | - |
| 16 | Renal disease, severe | 3 | Renal failure | - |
| 17 | HIV infection, no AIDS | 3 | - | - |
| 18 | Metastatic solid tumor | 6 | - | - |
| 19 | AIDS | 6 | - | - |

Table S2. Distribution by point of mCCI (absolute number)

| **mCCI** | **OKZ q4w** | **OKZ q2w** | **ADA** | **Placebo** |
| --- | --- | --- | --- | --- |
| *2* | *99* | *85* | *84* | *48* |
| 3 | 20 | 21 | 20 | 8 |
| 4 | 2 | 5 | 3 | 1 |
| 5 | 2 | 3 | 2 | 1 |
| 6 | 1 | 0 | 0 | 0 |
| 7 | 0 | 1 | 1 | 0 |
| *≥3* | *25* | *30* | *26* | *10* |

Table S3. Response rate at Week 12 and Week 24

|  | **OKZ q2w** | | **OKZ q4w** | | **ADA** | | **Placebo** | |
| --- | --- | --- | --- | --- | --- | --- | --- | --- |
|  | **NCC**  **N=348** | **CC**  **N=115** | **NCC**  **N=353** | **CC**  **N=124** | **NCC**  **N=352** | **CC**  **N=110** | **NCC**  **N=185** | **CC**  **N=58** |
| **CDAI ≤ 10** |  |  |  |  |  |  |  |  |
| Week 12 | 136 (39.1) | 41 (35.7) | 143 (40.5) | 36 (29.0) | 150 (42.6) | 34 (30.9) | 32 (17.3) | 11 (19.0) |
| Week 24 | 173 (49.7) | 48 (41.7) | 176 (49.9) | 52 (41.9) | 192 (54.5) | 43 (39.1) | 41 (22.2) | 18 (31.0) |
|  |  |  |  |  |  |  |  |  |
| **CDAI ≤ 2.8** |  |  |  |  |  |  |  |  |
| Week 12 | 28 ( 8.0) | 8 ( 7.0) | 33 ( 9.3) | 4 ( 3.2) | 32 ( 9.1) | 5 ( 4.5) | 4 ( 2.2) | 3 ( 5.2) |
| Week 24 | 40 (11.5) | 11 ( 9.6) | 46 (13.0) | 12 ( 9.7) | 55 (15.6) | 5 ( 4.5) | 8 ( 4.3) | 2 ( 3.4) |
|  |  |  |  |  |  |  |  |  |
| **SDAI ≤ 3.3** |  |  |  |  |  |  |  |  |
| Week 12 | 31 ( 8.9) | 11 ( 9.6) | 38 (10.8) | 7 ( 5.6) | 32 ( 9.1) | 4 ( 3.6) | 3 ( 1.6) | 3 ( 5.2) |
| Week 24 | 48 (13.8) | 13 (11.3) | 60 (17.0) | 14 (11.3) | 55 (15.6) | 5 ( 4.5) | 7 ( 3.8) | 2 ( 3.4) |
|  |  |  |  |  |  |  |  |  |
| **DAS28 (CRP) < 3.2** |  |  |  |  |  |  |  |  |
| Week 12 | 162 (46.6) | 50 (43.5) | 170 (48.2) | 48 (38.7) | 145 (41.2) | 32 (29.1) | 21 (11.4) | 10 (17.2) |
| Week 24 | 183 (52.6) | 58 (50.4) | 193 (54.7) | 62 (50.0) | 168 (47.7) | 42 (38.2) | 38 (20.5) | 14 (24.1) |
|  |  |  |  |  |  |  |  |  |
| **ACR50** |  |  |  |  |  |  |  |  |
| Week 12 | 149 (42.8) | 43 (37.4) | 160 (45.3) | 43 (34.7) | 148 (42.0) | 33 (30.0) | 28 (15.1) | 10 (17.2) |
| Week 24 | 181 (52.0) | 54 (47.0) | 181 (51.3) | 58 (46.8) | 178 (50.6) | 36 (32.7) | 38 (20.5) | 17 (29.3) |
|  |  |  |  |  |  |  |  |  |
| **ACR20** |  |  |  |  |  |  |  |  |
| Week 12 | 248 (71.3) | 81 (70.4) | 256 (72.5) | 83 (66.9) | 247 (70.2) | 62 (56.4) | 83 (44.9) | 25 (43.1) |
| Week 24 | 269 (77.3) | 78 (67.8) | 249 (70.5) | 90 (72.6) | 249 (70.7) | 70 (63.6) | 85 (45.9) | 28 (48.3) |

Table S4. Predicted probabilities with 95% CI from logistic regression by outcomes at Week 12

|  |  | **Model 1** | | | **Model 2** | | |
| --- | --- | --- | --- | --- | --- | --- | --- |
| **Outcomes** | **Treatment group** | **NCC** | **CC** | **p-value** | **NCC** | **CC** | **p-value** |
| **CDAI ≤ 10** | OKZ q2w | 39.1(34.1- 44.3) | 36.0 (27.7 - 45.2) | 0.583 | 37.8 (30.8 - 45.4) | 36.1 (26.5 - 46.9) | 0.636 |
|  | OKZ q4w | 40.5 (35.5 - 45.7) | 29.3 (21.9 - 37.9) | 0.173 | 39.8 (32.6 - 47.4) | 28.4 (20.0 - 38.7) | 0.168 |
|  | ADA | 42.6 (37.5 - 47.8) | 30.9 (23.0 - 40.1) | 0.171 | 41.9 (34.6 - 49.6) | 30.2 (21.3 - 41.0) | 0.170 |
|  | Placebo | 17.3 (12.5 - 23.4) | 19.0 (10.8 - 31.1) | 0.772 | 15.5 (10.4 - 22.5) | 17.6 (9.3 - 30.5) | 0.721 |
| **DAS28 (CRP) ≤ 3.2** | OKZ q2w | 46.6 (41.4 - 51.8) | 43.5 (34.7 - 52.7) | 0.194 | 48.7 (41.3 - 56.2) | 44.4 (34.2 - 55.0) | 0.129 |
|  | OKZ q4w | 48.2 (43.0 - 53.4) | 38.7 (30.6 - 47.5) | 0.063 | 50.2 (42.8 - 57.6) | 40.2 (30.6 - 50.6) | **0.045** |
|  | ADA | 41.2 (36.2 - 46.4) | 29.1 (21.4 - 38.2) | **0.033** | 42.8 (35.7 - 50.2) | 29.3 (20.7 - 39.7) | **0.019** |
|  | Placebo | 11.4 (7.5 - 16.8) | 17.2 (9.5 - 29.2) | 0.244 | 11.3 (7.1 - 17.5) | 18.2 (9.8 - 31.3) | 0.192 |
| **ACR50** | OKZ q2w | 42.8 (37.7 - 48.1) | 37.4 (29.0 - 46.6) | 0.407 | 46.7 (39.6 - 54.0) | 40.7 (31.0 - 51.3) | 0.438 |
|  | OKZ q4w | 45.3 (40.2 - 50.6) | 34.7 (26.8 - 43.5) | 0.190 | 48.7 (41.5 - 55.9) | 37.4 (28.2 - 47.6) | 0.207 |
|  | ADA | 42.0 (37.0 - 47.3) | 30.0 (22.2 - 39.2) | 0.144 | 45.7 (38.6 - 53.0) | 31.8 (22.9 - 42.3) | 0.133 |
|  | Placebo | 15.1 (10.7 - 21.0) | 17.2 (9.5 - 29.2) | 0.700 | 17.0 (11.6 - 24.4) | 18.8 (10.2 - 31.9) | 0.775 |

**Notes:** Model 1 = Crude model (without adjustment)

Model 2 = Adjusted for age and sex + duration of prior MTX use + duration of RA + baseline values

p-value for interaction terms in logistic regression is presented

Table S5. OR with 95% CI for for CC versus NCC groups; ADA and OKZ versus Placebo from logistic regression by outcomes at Week 12

|  |  | **Model 1** | | | | **Model 2** | | | |
| --- | --- | --- | --- | --- | --- | --- | --- | --- | --- |
| **Outcomes** | **Treatment group** | **OR (95% CI)**  **vs. Placebo** | **p-value** | **OR**  **CC vs NCC**  **(95% CI)** | **p-value*** | **OR (95% CI)**  **vs. Placebo** | **p-value** | **OR**  **CC vs NCC**  **(95% CI)** | **p-value*** |
| **CDAI ≤ 10** | **ADA** | 3.55 (2.32 - 5.56) | <0.001 | 0.54 (0.23 - 1.34) | 0.171 | 3.93 (2.51 - 6.29) | <0.001 | 0.52 (0.21 - 1.36) | 0.170 |
|  | **OKZ q2w** | 3.07 (2.00 - 4.81) | <0.001 | 0.78 (0.33 - 1.93) | 0.583 | 3.31 (2.11 - 5.32) | <0.001 | 0.80 (0.32 - 2.08) | 0.636 |
|  | **OKZ q4w** | 3.26 (2.13 - 5.10) | <0.001 | 0.54 (0.23 - 1.34) | 0.173 | 3.59 (2.30 - 5.76) | <0.001 | 0.52 (0.21 - 1.36) | 0.168 |
| **DAS28 (CRP) ≤ 3.2** | **ADA** | 5.47 (3.38 - 9.25) | <0.001 | 0.36 (0.14 - 0.95) | **0.033** | 5.86 (3.58 - 10.01) | <0.001 | 0.32 (0.12 - 0.85) | **0.019** |
|  | **OKZ q2w** | 6.80 (4.20 - 11.50) | <0.001 | 0.54 (0.22 - 1.40) | 0.194 | 7.44 (4.55 - 12.72) | <0.001 | 0.48 (0.19 - 1.27) | 0.129 |
|  | **OKZ q4w** | 7.25 (4.49 - 12.25) | <0.001 | 0.42 (0.17 - 1.08) | 0.063 | 7.90 (4.84 - 13.46) | <0.001 | 0.38 (0.15 - 1.00) | **0.045** |
| **ACR50** | **ADA** | 4.07 (2.62 - 6.51) | <0.001 | 0.51 (0.21 - 1.30) | 0.144 | 4.09 (2.63 - 6.56) | <0.001 | 0.49 (0.20 - 1.27) | 0.133 |
|  | **OKZ q2w** | 4.20 (2.70 - 6.72) | <0.001 | 0.68 (0.28 - 1.74) | 0.407 | 4.26 (2.73 - 6.85) | <0.001 | 0.70 (0.29 - 1.79) | 0.438 |
|  | **OKZ q4w** | 4.65 (2.99 - 7.43) | <0.001 | 0.55 (0.23 - 1.39) | 0.190 | 4.62 (2.97 - 7.41) | <0.001 | 0.56 (0.23 - 1.42) | 0.207 |

**Notes:** Model 1 = Crude model (without adjustment)

Model 2 = Adjusted for age and sex + duration of prior MTX use + duration of RA + baseline values

*p-value for interaction terms in logistic regression is presented

Table S6. Predicted probabilities with 95% CI from logistic regression by outcomes at Week 24

|  |  | **Model 1** | | | **Model 2** | | |
| --- | --- | --- | --- | --- | --- | --- | --- |
| **Outcomes** | **Treatment group** | **NCC** | **CC** | **p-value** | **NCC** | **CC** | **p-value** |
| **CDAI ≤ 10** | OKZ q2w | 49.7 (44.5 - 54.9) | 42.1 (33.4 - 51.3) | 0.055 | 56.4 (48.9 - 63.6) | 46.4 (35.9 - 57.2) | 0.063 |
|  | OKZ q4w | 49.9 (44.7 - 55.1) | 42.3 (33.9 - 51.2) | 0.054 | 55.9 (48.4 - 63.2) | 45.4 (35.1 - 56.1) | 0.055 |
|  | ADA | 54.5 (49.3 - 59.7) | 39.1 (30.4 - 48.5) | **0.007** | 60.4 (52.9 - 67.4) | 44.3 (33.8 - 55.3) | **0.015** |
|  | Placebo | 22.2 (16.8 - 28.7) | 31.0 (20.5 - 44.0) | 0.171 | 24.3 (17.5 - 32.8) | 32.1 (20.2 - 46.9) | 0.281 |
| **CDAI ≤ 2.8** | OKZ q2w | 11.5 (8.5 - 15.3) | 9.6 (5.4 - 16.5) | 0.973 | 14.0 (9.5 - 20.2) | 10.2 (5.3 - 18.8) | 0.789 |
|  | OKZ q4w | 13.0 (9.9 - 17.0) | 9.7 (5.6 - 16.3) | 0.909 | 15.0 (10.2 - 21.4) | 12.0 (6.6 - 20.8) | 0.721 |
|  | ADA | 15.6 (12.2 - 19.8) | 4.5 (1.9 - 10.5) | 0.231 | 18.3 (12.8 - 25.3) | 5.3 (2.1 - 12.6) | 0.548 |
|  | Placebo | 4.3 (2.2 - 8.4) | 3.4 (0.9 - 12.8) | 0.770 | 4.0 (1.7 - 8.9) | 2.1 (0.3 - 13.5) | 0.543 |
| **SDAI ≤ 3.3** | OKZ q2w | 13.8 (10.6 - 17.8) | 11.3 (6.7 - 18.5) | 0.882 | 17.1 (12.0 - 23.8) | 12.0 (6.6 - 20.9) | 0.940 |
|  | OKZ q4w | 17.0 (13.4 - 21.3) | 11.3 (6.8 - 18.2) | 0.665 | 20.4 (14.7 - 27.7) | 13.8 (7.9 - 22.9) | 0.980 |
|  | ADA | 15.6 (12.2 - 19.8) | 4.5 (1.9 - 10.5) | 0.183 | 18.8 (13.4 - 25.7) | 5.3 (2.1 - 12.4) | 0.443 |
|  | Placebo | 3.8 (1.8 - 7.7) | 3.4 (0.9 - 12.8) | 0.906 | 3.3 (1.3 - 8.1) | 2.1 (0.3 - 13.4) | 0.653 |
| **DAS28 (CRP) ≤ 3.2** | OKZ q2w | 52.6 (47.3 - 57.8) | 50.4 (41.4 - 59.5) | 0.481 | 56.8 (49.4 - 63.8) | 52.5 (42.0 - 62.8) | 0.396 |
|  | OKZ q4w | 54.7 (49.4 - 59.8) | 50.0 (41.3 - 58.7) | 0.339 | 57.9 (50.6 - 64.9) | 53.3 (43.0 - 63.3) | 0.370 |
|  | ADA | 47.7 (42.6 - 53.0) | 38.2 (29.6 - 47.6) | 0.155 | 50.9 (43.6 - 58.2) | 39.7 (29.8 - 50.5) | 0.136 |
|  | Placebo | 20.5 (15.3 - 27.0) | 24.1 (14.8 - 36.7) | 0.560 | 21.1 (15.0 - 29.0) | 24.6 (14.5 - 38.5) | 0.601 |
| **ACR50** | OKZ q2w | 52.0 (46.8 - 57.2) | 47.0 (38.0 - 56.1) | 0.094 | 58.6 (51.4 - 65.4) | 52.5 (42.1 - 62.6) | 0.102 |
|  | OKZ q4w | 51.3 (46.1 - 56.5) | 46.8 (38.2 - 55.6) | 0.103 | 57.2 (50.1 - 64.1) | 51.9 (41.9 - 61.8) | 0.117 |
|  | ADA | 50.6 (45.4 - 55.8) | 32.7 (24.6 - 42.0) | **0.003** | 56.7 (49.5 - 63.6) | 36.6 (27.1 - 47.3) | **0.003** |
|  | Placebo | 20.5 (15.3 - 27.0) | 29.3 (19.1 - 42.2) | 0.166 | 24.9 (18.1 - 33.2) | 33.5 (21.7 - 47.8) | 0.226 |

**Notes:** Model 1 = Crude model (without adjustment)

Model 2 = Adjusted for age and sex + duration of prior MTX use + duration of RA + baseline values

p-value for interaction terms in logistic regression is presented

Table S7. OR with 95% CI for CC versus NCC groups; ADA and OKZ versus Placebo from logistic regression by outcomes at Week 24

|  |  | **Model 1** | | | | **Model 2** | | | |
| --- | --- | --- | --- | --- | --- | --- | --- | --- | --- |
|  |  | **OR (95% CI)**  **vs. Placebo** | **p-value** | **OR**  **CC vs NCC**  **(95% CI)** | **p-value*** | **OR (95% CI)**  **vs. Placebo** | **p-value** | **OR**  **CC vs NCC***  **(95% CI)** | **p-value*** |
| **CDAI ≤ 10** | **ADA** | 4.21 (2.83 - 6.38) | <0.001 | 0.34 (0.15 - 0.75) | **0.007** | 4.74 (3.12 - 7.35) | <0.001 | 0.35 (0.15 - 0.83) | **0.015** |
|  | **OKZ q2w** | 3.47 (2.33 - 5.26) | <0.001 | 0.47 (0.21 - 1.03) | 0.055 | 4.03 (2.64 - 6.25) | <0.001 | 0.45 (0.20 - 1.06) | 0.063 |
|  | **OKZ q4w** | 3.49 (2.35 - 5.28) | <0.001 | 0.47 (0.22 - 1.02) | 0.054 | 3.95 (2.60 - 6.11) | <0.001 | 0.44 (0.20 - 1.03) | 0.055 |
| **CDAI ≤ 2.8** | **ADA** | 4.10 (2.02 - 9.49) | <0.001 | 0.33 (0.06 - 2.61) | 0.231 | 5.43 (2.46 - 14.39) | <0.001 | 0.49 (0.06 - 10.35) | 0.548 |
|  | **OKZ q2w** | 2.87 (1.38 - 6.74) | 0.008 | 1.03 (0.21 - 7.70) | 0.973 | 3.96 (1.76 - 10.61) | 0.002 | 1.36 (0.19 - 27.83) | 0.789 |
|  | **OKZ q4w** | 3.32 (1.61 - 7.73) | 0.002 | 0.90 (0.19 - 6.70) | 0.909 | 4.28 (1.92 - 11.41) | 0.001 | 1.51 (0.22 - 30.46) | 0.721 |
| **SDAI ≤ 3.3** | **ADA** | 4.71 (2.24 - 11.55) | <0.001 | 0.28 (0.05 - 2.30) | 0.183 | 6.69 (2.87 - 19.56) | <0.001 | 0.40 (0.05 - 8.52) | 0.443 |
|  | **OKZ q2w** | 4.07 (1.92 - 10.03) | <0.001 | 0.88 (0.18 - 6.55) | 0.882 | 5.98 (2.54 - 17.57) | <0.001 | 1.09 (0.15 - 22.43) | 0.940 |
|  | **OKZ q4w** | 5.21 (2.49 - 12.74) | <0.001 | 0.68 (0.14 - 5.07) | 0.665 | 7.43 (3.19 - 21.67) | <0.001 | 1.03 (0.14 - 20.99) | 0.980 |
| **DAS28 (CRP) ≤ 3.** | **ADA** | 3.53 (2.36 - 5.40) | <0.001 | 0.55 (0.24 - 1.27) | 0.155 | 3.87 (2.55 - 6.00) | <0.001 | 0.52 (0.22 - 1.25) | 0.136 |
|  | **OKZ q2w** | 4.29 (2.86 - 6.56) | <0.001 | 0.75 (0.33 - 1.72) | 0.481 | 4.90 (3.21 - 7.61) | <0.001 | 0.69 (0.30 - 1.65) | 0.396 |
|  | **OKZ q4w** | 4.67 (3.11 - 7.13) | <0.001 | 0.67 (0.30 - 1.54) | 0.339 | 5.14 (3.38 - 7.97) | <0.001 | 0.68 (0.30 - 1.61) | 0.370 |
| **ACR50** | **ADA** | 3.96 (2.64 - 6.04) | <0.001 | 0.30 (0.13 - 0.67) | **0.003** | 3.95 (2.62 - 6.04) | <0.001 | 0.29 (0.13 - 0.66) | **0.003** |
|  | **OKZ q2w** | 4.19 (2.80 - 6.41) | <0.001 | 0.51 (0.23 - 1.14) | 0.094 | 4.27 (2.84 - 6.56) | <0.001 | 0.51 (0.23 - 1.15) | 0.102 |
|  | **OKZ q4w** | 4.07 (2.72 - 6.22) | <0.001 | 0.52 (0.24 - 1.15) | 0.103 | 4.04 (2.69 - 6.18) | <0.001 | 0.53 (0.24 - 1.18) | 0.117 |

**Notes:** Model 1 = Crude model (without adjustments)

Model 2 = Adjusted for age and sex + duration of prior MTX use + duration of RA + baseline values

* p-value for interaction terms in logistic regression is presented

Table S8. Missing data at Week 12 and Week 24

|  | **OKZ q2w** | | **OKZ q4w** | | **ADA** | | **Placebo** | |
| --- | --- | --- | --- | --- | --- | --- | --- | --- |
|  | **NCC**  **N=348** | **CC**  **N=115** | **NCC**  **N=353** | **CC**  **N=124** | **NCC**  **N=352** | **CC**  **N=110** | **NCC**  **N=185** | **CC**  **N=58** |
| **CDAI ≤ 10** |  |  |  |  |  |  |  |  |
| Week 12 | 32 ( 9.2) | 14 (12.2) | 39 (11.0) | 12 ( 9.7) | 43 (12.2) | 18 (16.4) | 31 (16.8) | 7 (12.1) |
| Week 24 | 67 (19.3) | 27 (23.5) | 67 (19.0) | 22 (17.7) | 72 (20.5) | 24 (21.8) | 56 (30.3) | 14 (24.1) |
|  |  |  |  |  |  |  |  |  |
| **CDAI ≤ 2.8** |  |  |  |  |  |  |  |  |
| Week 12 | 32 ( 9.2) | 14 (12.2) | 39 (11.0) | 12 ( 9.7) | 43 (12.2) | 18 (16.4) | 31 (16.8) | 7 (12.1) |
| Week 24 | 67 (19.3) | 27 (23.5) | 67 (19.0) | 22 (17.7) | 72 (20.5) | 24 (21.8) | 56 (30.3) | 14 (24.1) |
|  |  |  |  |  |  |  |  |  |
| **SDAI ≤ 3.3** |  |  |  |  |  |  |  |  |
| Week 12 | 31 ( 8.9) | 13 (11.3) | 38 (10.8) | 12 ( 9.7) | 43 (12.2) | 18 (16.4) | 30 (16.2) | 7 (12.1) |
| Week 24 | 72 (20.7) | 29 (25.2) | 72 (20.4) | 21 (16.9) | 71 (20.2) | 25 (22.7) | 57 (30.8) | 13 (22.4) |
|  |  |  |  |  |  |  |  |  |
| **DAS28 (CRP) < 3.2** |  |  |  |  |  |  |  |  |
| Week 12 | 31 ( 8.9) | 13 (11.3) | 38 (10.8) | 12 ( 9.7) | 43 (12.2) | 18 (16.4) | 30 (16.2) | 7 (12.1) |
| Week 24 | 69 (19.8) | 28 (24.3) | 69 (19.5) | 20 (16.1) | 69 (19.6) | 25 (22.7) | 56 (30.3) | 12 (20.7) |
|  |  |  |  |  |  |  |  |  |
| **ACR50** |  |  |  |  |  |  |  |  |
| Week 12 | 31 ( 8.9) | 13 (11.3) | 38 (10.8) | 12 ( 9.7) | 43 (12.2) | 18 (16.4) | 30 (16.2) | 7 (12.1) |
| Week 24 | 68 (19.5) | 26 (22.6) | 66 (18.7) | 21 (16.9) | 72 (20.5) | 24 (21.8) | 57 (30.8) | 13 (22.4) |
